# Supplementary material for: Predicting visual function by interpreting a neuronal wiring diagram
Source: Nature. 2024 Oct 2;634(8032):113–23. doi: 10.1038/s41586-024-07953-5 (PMC11446822; doi:10.1038/s41586-024-07953-5)
Supplement: Supplementary file 6 — CRF and ERF predictions for individual TmY4 and TmY9 cells. Analogous to Supplementary Data 3, but for TmY target types. Shown are the top four monosynaptic pathways, the strongest pathway passing through each of the top ten intermediary types (ranking from Extended Data Fig. 7), and the trisynaptic pathway Tm1–TmY–Dm3–TmY (see the section entitled Prediction of spatial normalization). [file 41586_2024_7953_MOESM6_ESM.zip › DataS4/TmY9qΓèÑ/720575940629470455.pdf]

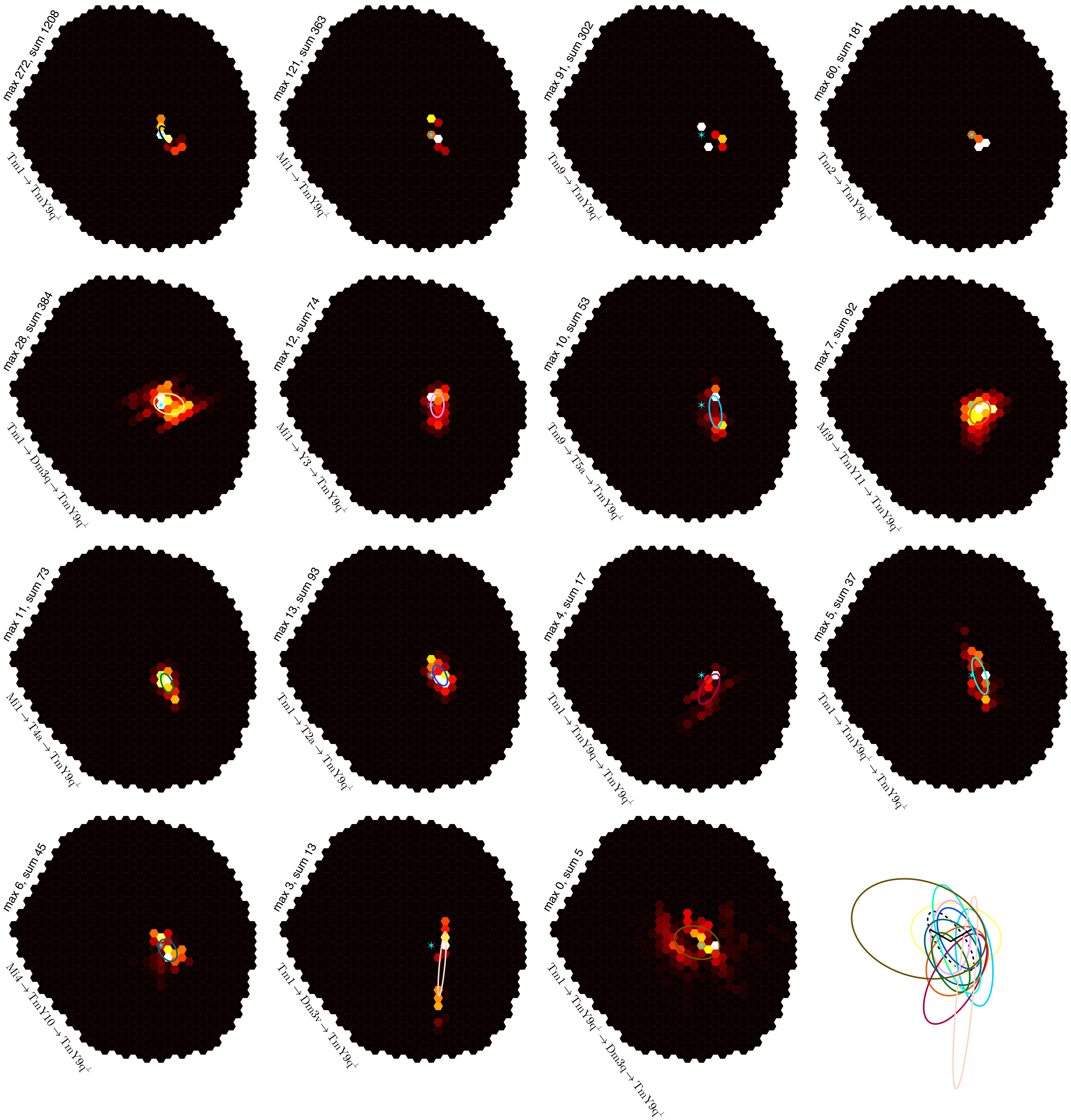

max 272, sum 1208
Tm1 → TmY9q⊥
max 121, sum 363
Mi1 → TmY9q⊥
max 91, sum 302
Tm9 → TmY9q⊥
max 60, sum 181
Tm2 → TmY9q⊥
max 28, sum 384
Tm1 → Dm3q → TmY9q⊥
max 12, sum 74
Mi1 → Y3 → TmY9q⊥
max 10, sum 53
Tm9 → T5a → TmY9q⊥
max 7, sum 92
Mi9 → TmY11 → TmY9q⊥
max 11, sum 73
Mi1 → T4a → TmY9q⊥
max 13, sum 93
Tm1 → T2a → TmY9q⊥
max 4, sum 17
Tm1 → TmY9q → TmY9q⊥
max 5, sum 37
Tm1 → TmY9q⊥ → TmY9q⊥
max 6, sum 45
Mi4 → TmY10 → TmY9q⊥
max 3, sum 13
Tm1 → Dm3v → TmY9q⊥
max 0, sum 5
Tm1 → TmY9q⊥ → Dm3q → TmY9q⊥
